# A Survival Case in a Severe Amlodipine Intoxication

**DOI:** 10.1155/2013/842606

**Published:** 2013-04-03

**Authors:** T. El Houari, I. Haddiya, N. El Ouafi, Z. Bazid

**Affiliations:** ^1^Department of Cardiology, Faculty of Medicine, University Mohamed First, Oujda, Morocco; ^2^Department of Nephrology, Faculty of Medicine, University Mohamed First, Oujda, Morocco

## Abstract

Calcium channel blockers (CCBs) are prescribed in a wide variety of cardiovascular conditions. Nevertheless, they remain a major cause of cardiovascular drug overdose that often leads to a lethal outcome. We report the case of an intoxication with amlodipine, which caused severe hypotension, in a young woman. The patient was initially treated with fluids, calcium gluconate, and Dobutamine without effect. She then received hyperinsulinemia euglycemia therapy. A rise in blood pressure (BP) was observed two hours after insulin was started. The next day, the insulin infusion was stopped and seven days later the patient was discharged from the hospital after psychiatric consultation. The positive inotropic effect of insulin therapy in our patient supports previous findings that suggest its use as a first-line therapy in the management of CCBs overdose.

## 1. Introduction

Calcium channel blockers (CCBs) are the first cause of cardiovascular drug overdose death [1]. In the 28th Annual Report of the American Association of Poison Control Centers, cardiovascular drugs caused 128 cases of fatalities with 24 deaths (18.75%) due to amlodipine [2].

Amlodipine is a dihydropyridine calcium channel blocking agent used in the treatment of essential hypertension and angina pectoris. It is prescribed with a daily dose of 5–10 mg. Unlike other calcium channel blockers, amlodipine has a very low metabolic clearance with the advantage of using a once-daily dosage to maintain a near-constant plasma concentration [3].

There are several cases of amlodipine overdoses reported with several of them having a lethal outcome. We report a survival case in a severe amlodipine intoxication.

## 2. Case Report

A 27-year-old woman with no known illnesses or history of psychiatric disorders was admitted approximately 6 hours after attempting suicide by ingesting 150 mg of amlodipine. She was newly married and had a conjugal conflict. 

On admission, the patient was conscious but lethargic with cold extremities. Her blood pressure was 75/49 mmHg and the pulse rate was 105** **bpm. She received gastric lavage and still complained of nausea. 

The patient was started on intravenous fluids and transferred to the intensive care unit. On physical examination, the patient was slightly lethargic and had the following vital signs: systolic blood pressure, 80 mmHg; pulse, 125 bpm; respiratory rate, 26 breaths/min; and temperature, 36.5°C, pulse oximetry displayed 98% oxygen saturation under oxygen supplement. The head and neck exams were unremarkable. The cardiac examination found normal heart sounds without any murmurs. The lungs were clear to auscultation. The extremities were cold, with neither edema nor cyanosis. 

The laboratory investigations showed a white blood cell count of 12,400/mm^3^ with 75% neutrophils, hemoglobin of 10.3 g/dL, and platelet count of 282,000/mm^3^. The electrolyte rates were sodium, 134 mEq/L; potassium, 3.8 mEq/L; bicarbonate, 16 mEq/L; blood urea, 18 mg/dL; creatinine, 2.3 mg/dL; and glucose, 85 mg/dL. The initial electrocardiogram showed a sinus tachycardia at 120 bpm with normal QRS, nonspecific ST segment, and T wave abnormalities. The chest radiograph was normal.

Despite the intravenous fluid infusion, 2000 mL crystalloids, the mean arterial blood pressure remained below 55 mmHg. Simultaneously, two grams of calcium gluconate were administered, without the improvement of arterial blood pressure. Dobutamine infusion was also started in an effort to increase the patient's inotropy. However, the patient's status remained unchanged at 15 *μ*g/kg/min. Moreover, an insulin infusion was initiated at 0.5 IU/kg/h. Blood sugar levels were measured every hour and glucose administrated whenever they reached rates lower than 100 mg/dL. Two hours later, the patient's blood pressure increased to 90/45 mmHg. Over the next four hours, there was a sustained increase in blood pressure to 100/50 mmHg. Dobutamine dose was progressively reduced, and then suspended over the next day. The mean blood pressure remained above 90 mmHg. Twenty four hours later, the insulin infusion was stopped. Seven days later the patient was discharged from the hospital after psychiatric consultation.

## 3. Comment

Calcium channel blockers (CCBs) have been increasingly used for a variety of cardiovascular conditions, such as hypertension, angina, and supraventricular tachyarrhythmias. Dihydropyridine CCBs have a predominant effect on vascular smooth muscle cells with little effect on cardiac pacemaker cells or contractility [4]. That explains why our patient presented with a refractory hypotension without cardiac conduction defects. Nonetheless, when compared to the first generation of CCBs, amlodipine overdose may cause a longer duration of toxicity. 

Calcium efficacy to improve hypotension, contractility, and conduction is inconstantly successful in patients with CCBs overdose. In this case, we used two grams of calcium gluconate without any effect. 

Some authors reported the use of vasopressin analogues in cases of hypotension due to sustained released calcium antagonists. In fact, by using Terlipressin, hemodynamic stabilization could be achieved and the patient survived [5].

Hyperinsulinemic euglycemia in CCBs overdose was benefic in several case reports. This approach was usually started after calcium gluconate and vasoactives drugs failure. Insulin effect is due to increasing plasma levels of ionized calcium, improving the hyperglycemic acidotic state, myocardial utilization of carbohydrates, and exerting its own independent inotropic effect [6]. In our case this method was successful. 

The absence of standardized dosages of calcium and dextrose-insulin infusion makes the efficacy of these approaches fluctuate. Usually insulin is started after vasopressors and calcium failure. These findings suggest that dextrose-insulin infusions should be considered as a first-line therapy in the management of CCBs overdose.
